# Supplementary material for: Poly(4-styrenesulfonate)-induced sulfur vacancy self-healing strategy for monolayer MoS2 homojunction photodiode
Source: Nat Commun. 2017 Jun 22;8:15881. doi: 10.1038/ncomms15881 (PMC5489680; doi:10.1038/ncomms15881)
Supplement: Supplementary Information [file ncomms15881-s1.pdf]

Type of file: PDF
Size of file: 0 KB
Title of file for HTML: Supplementary Information
Description: Supplementary Figures, Supplementary Table, Supplementary Notes, Supplementary Methods and Supplementary References

Type of file: PDF
Size of file: 0 KB
Title of file for HTML: Peer Review File
Description:

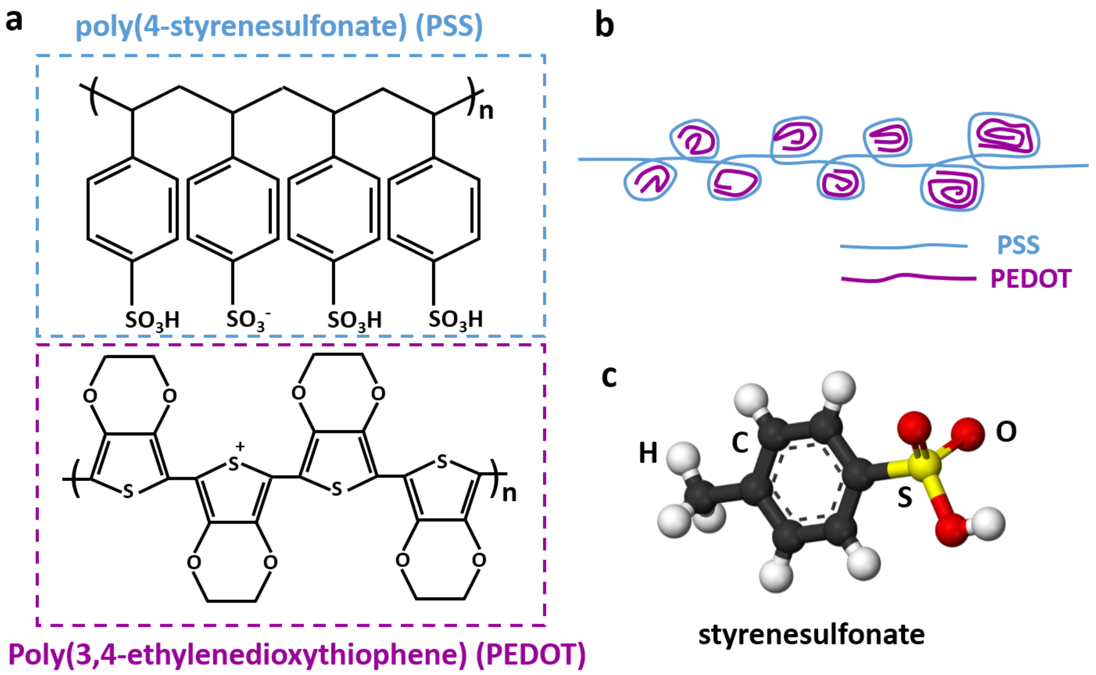

Supplementary Figure 1. (**a**) Chemical structure of Poly(3,4-ethylenedioxythiophene): poly(4-styrenesulfonate) (PEDOT:PSS). (**b**) Conformations of PEDOT:PSS. The thin and thick curves stand for PSS and PEDOT chains, respectively. The current suggested model for the morphology of PEDOT:PSS solid films is that it consists of grains with a highly conductive PEDOT-rich core and a sulfonic acid PSS-rich shell. (**c**) Three-dimensional (3D) representation of the structure of styrenesulfonate. Styrenesulfonate is monomer of the poly(4-styrenesulfonate) (PSS).

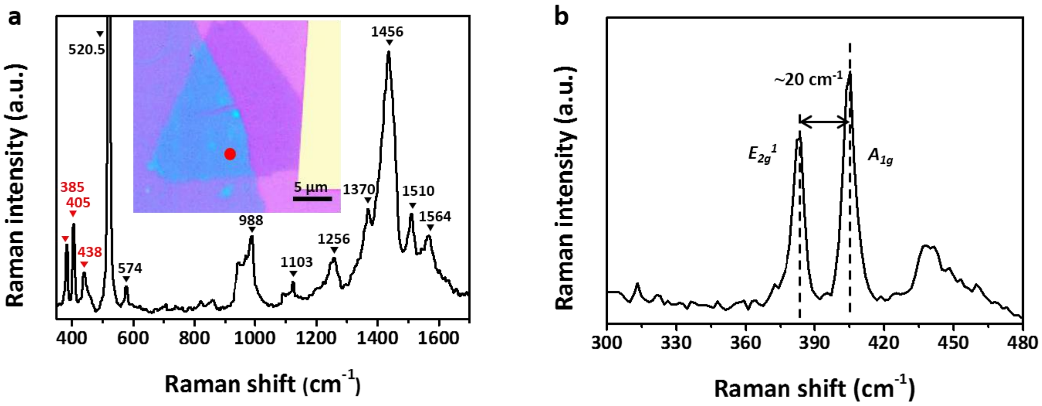

Supplementary Figure 2. (**a**) Raman spectrum of the overlapped area between MoS_2 and PEDOT:PSS electrode acquired from the red dot highlighted in inset. (**b**) The partial enlarged view of monolayer MoS_2 Raman spectrum.

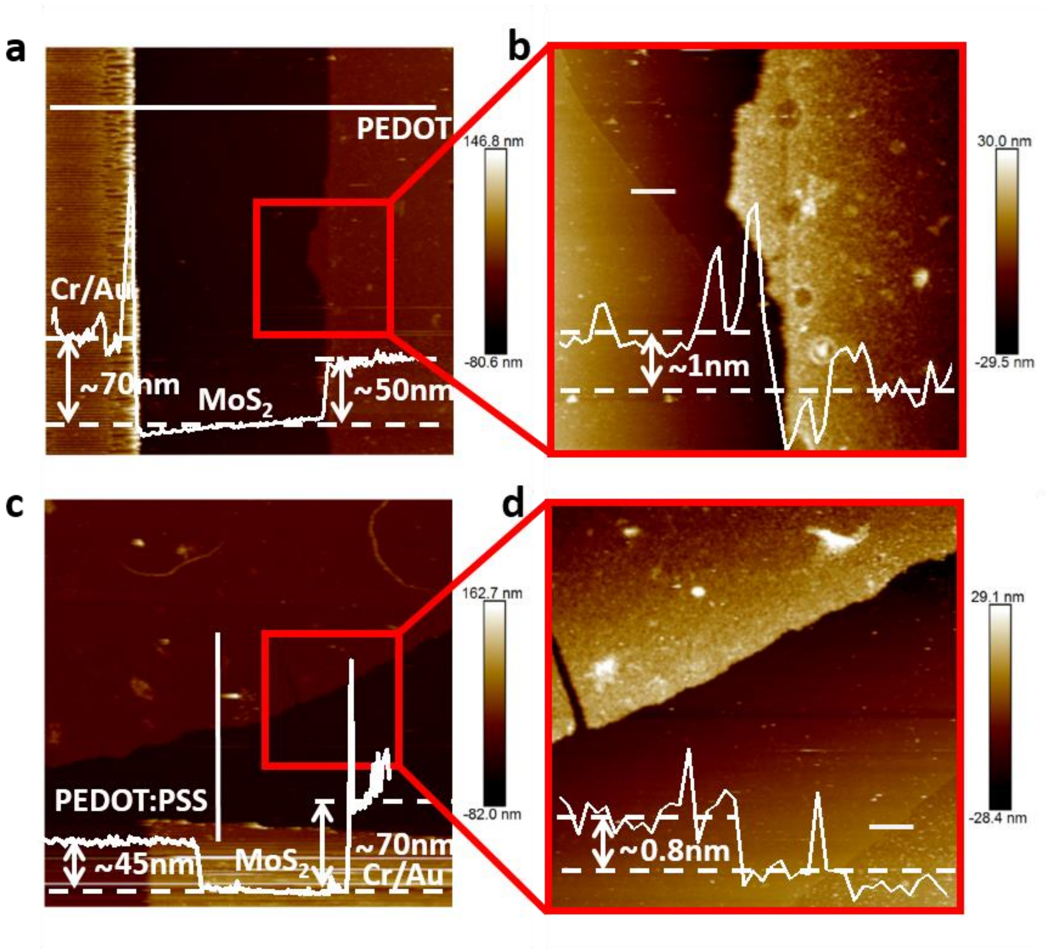

Supplementary Figure 3. (**a-b**) Atomic force microscopy image of the device B without PSS treatment. It shows that the PEDOT, Cr/Au and MoS_2 film are ~50 nm, ~70 nm and ~1 nm thick, respectively. **b** is the partial enlarged view of the red square shown in **a**. (**c-d)** AFM image of the device A_1 with PSS treatment. It shows that the PEDOT:PSS, Cr/Au and MoS_2 film are ~45 nm, ~70 nm and ~0.8 nm thick, respectively. **d** is the partial enlarged view of the red square **c**. In the AFM image, we hardly find out the ultrathin MoS_2 film on same scale like 2D surface potential image, unless it is enlarged to larger multiples.

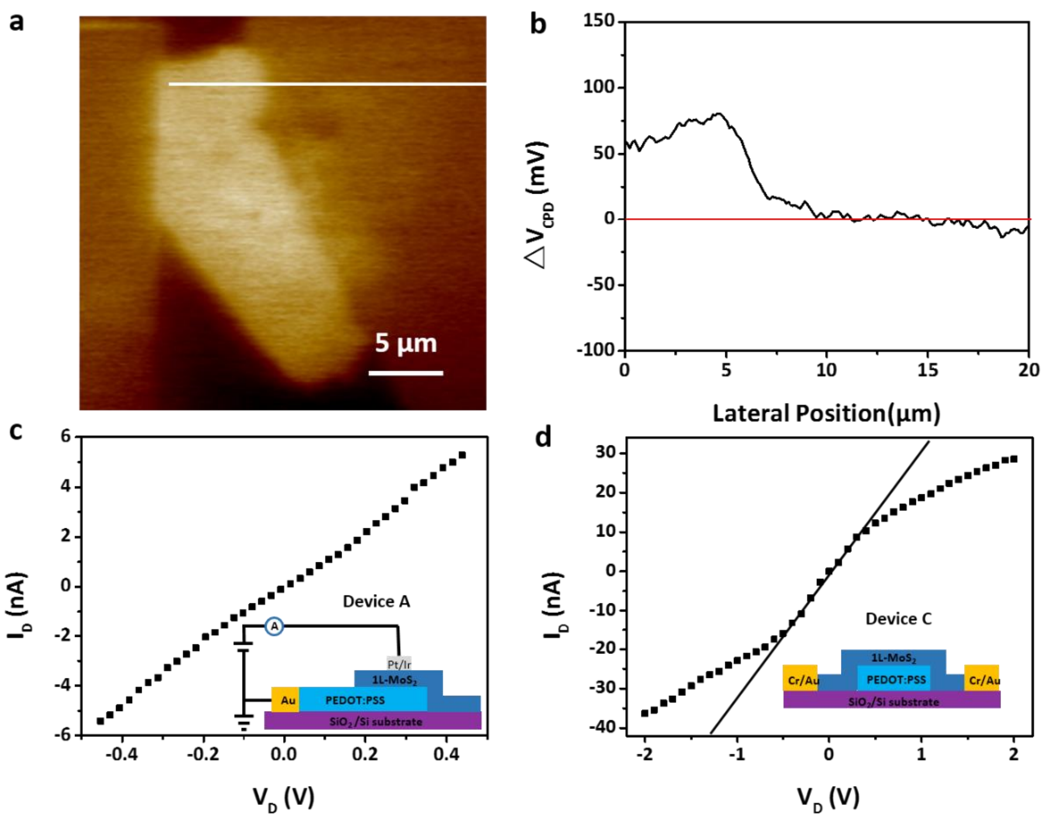

Supplementary Figure 4. (**a**) 2D surface potential images of the monolayer MoS_2 homojunction device A_1. (**b**) The corresponding work functions at the white line position shown in **a**. the data suggests compared to the potential barrier between the as-grown and self-healing MoS_2, the potential barrier between the self-healed MoS_2 and PEDOT:PSS electrode can be negligible. (**c**) I-V characteristic of the vertical MoS_2/PEDOT:PSS junction of the MoS_2 homojunction diode by AFM. This result again confirms that conclusion there isn't potential barrier between the self-healed MoS_2 and PEDOT:PSS electrode[1,2]. (**d**) I-V characteristic of the n-p-n MoS_2 monolayer FET and the only difference from typical MoS_2 FET is that monolayer MoS_2 lays across the PEDOT:PSS film in this transistor shown in the bottom right inset. PSS-induced sulfur vacancy self-healing give rise to rectifying characteristic similar to that of double Schottky or n-p-n type devices.

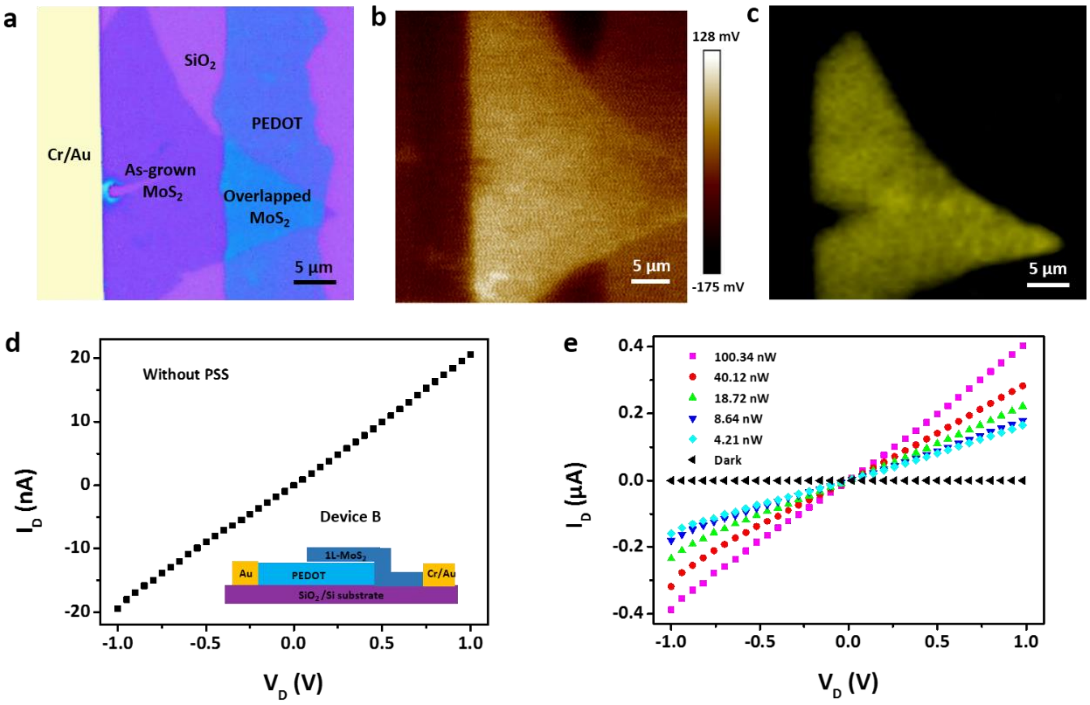

Supplementary Figure 5. (a) optical microscopy images of the MoS_2 monolayer FET (device B) with PEDOT electrode without PSS (methods). (**b**) corresponding 2D surface potential images. (**c**) PL spectrum intensity mapping. When the surface PSS of PEDOT:PSS electrode is removed, there is no surface potential (PL spectrum intensity) variation between the overlapped and as-grown MoS_2 triangle in **b** (**c**). (**d**) Output characteristic of the MoS_2 monolayer FET with PEDOT electrode without PSS. Comparing with the surface potential images, PL spectrum intensity mapping and output curve shown in Fig. 1c-d and Fig. 3b, it can be concluded that PSS is an essential element in the process of forming our monolayer MoS_2 homojunction. (**e**) Output characteristics of device B in dark and under different incident light intensity. The device B operates as an enhancement-mode transistor. Increasing illumination levels result in enhanced current due to electron-hole pair generation by light absorption in the direct bandgap of monolayer MoS_2.

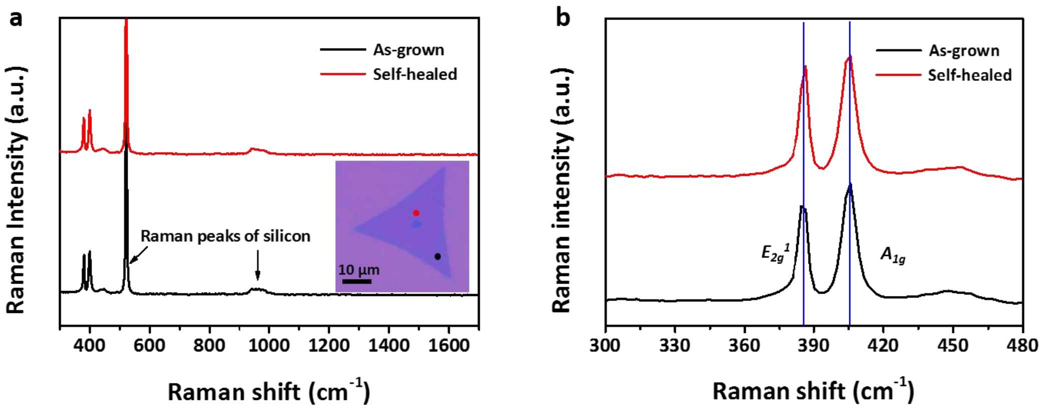

Supplementary Figure 6. (**a**) Raman spectrum of the CVD monolayer MoS_2 before and after PSS-induced SVSH. Raman spectrums acquired from different regions highlighted in inset. (**b)** The partial enlarged view of monolayer MoS_2 Raman spectrum in **a**.

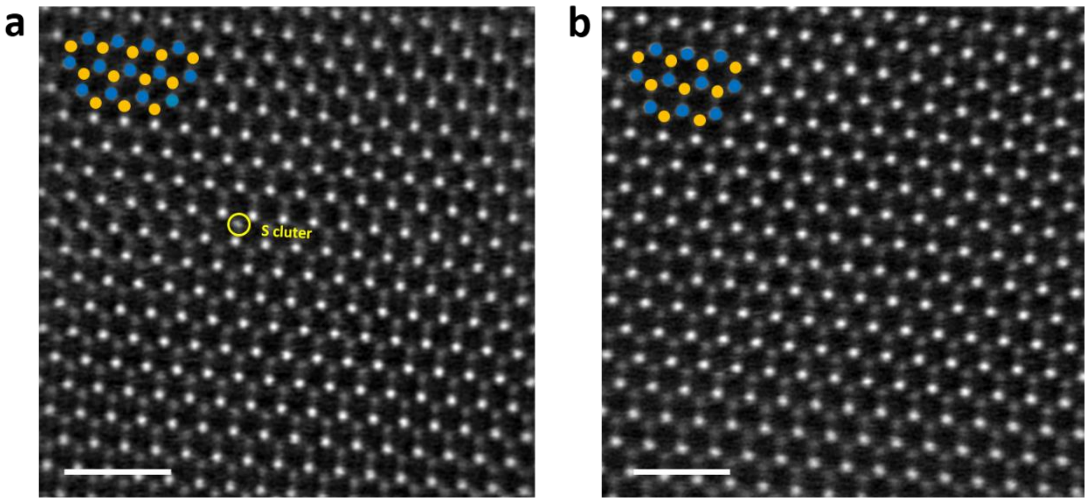

Supplementary Figure 7. (a-b) STEM image of the as-grown and self-healed MoS_2. The cyan and yellow dots indicate the Mo and S atoms, respectively. the atom with the yellow circle is the sulfur adatom cluster. Scale bar, 1 nm.

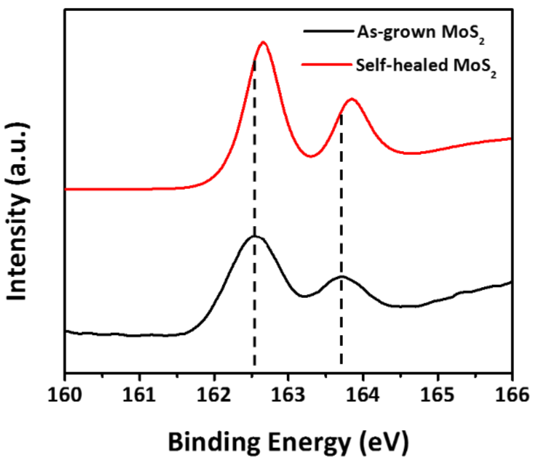

Supplementary Figure 8. High-resolution XPS for S 2p before (top) and after (bottom) PSS treatment of MoS_2.

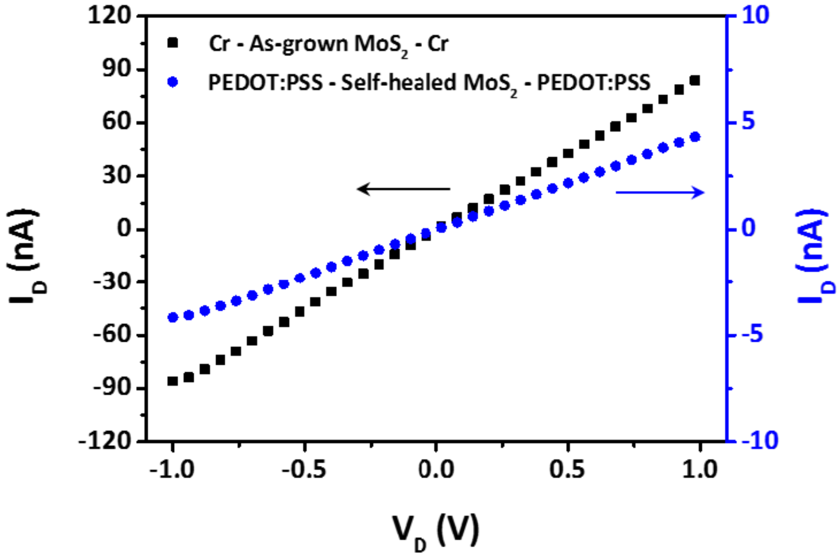

Supplementary Figure 9. Output characteristic of the typical MoS_2 FET with Cr/Au or PEDOT:PSS electrodes. Ohmic characteristics are all observed among the following two contact types: as-grown MoS_2 and Cr/Au electrode, and self-healed MoS_2 and PEDOT: PSS electrode.

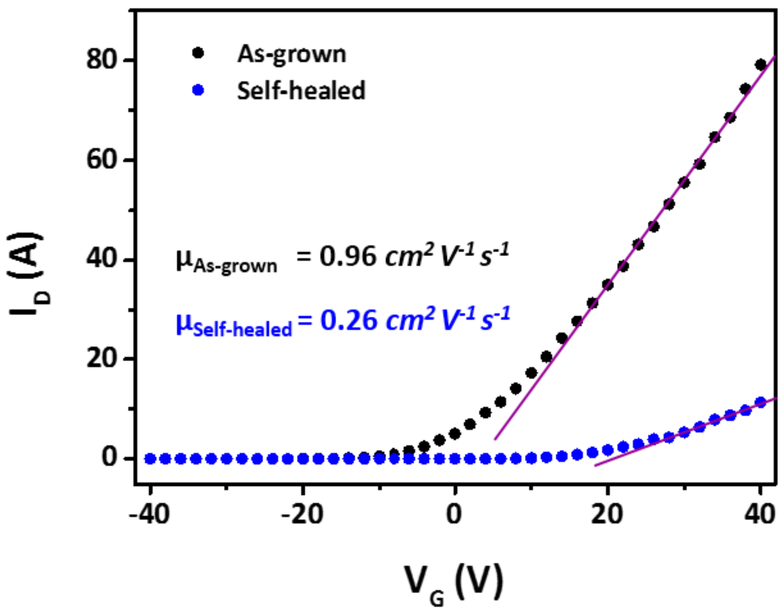

Supplementary Figure 10. Transfer characteristics of a monolayer MoS_2 transistor both before and after PSS-induced sulfur vacancy self-healing.

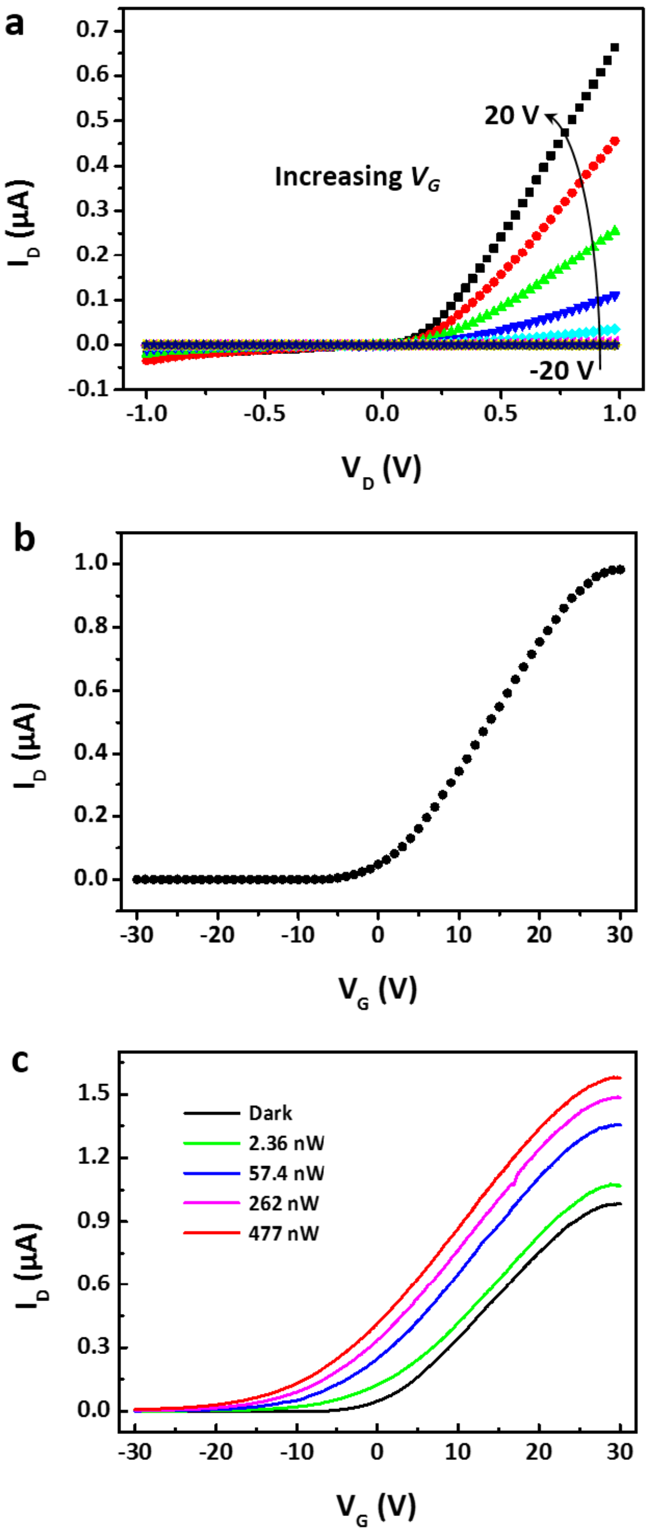

Supplementary Figure 11. (**a**) Output characteristics of the homojunction at various V_G levels between 20 and -20V, along steps of 5V. (**b**) Transfer characteristic of the device A_2 at V_D=1V. (**c**) Dependence on gate voltage of the drain current in dark and under different incident light intensity, at V_D=1V.

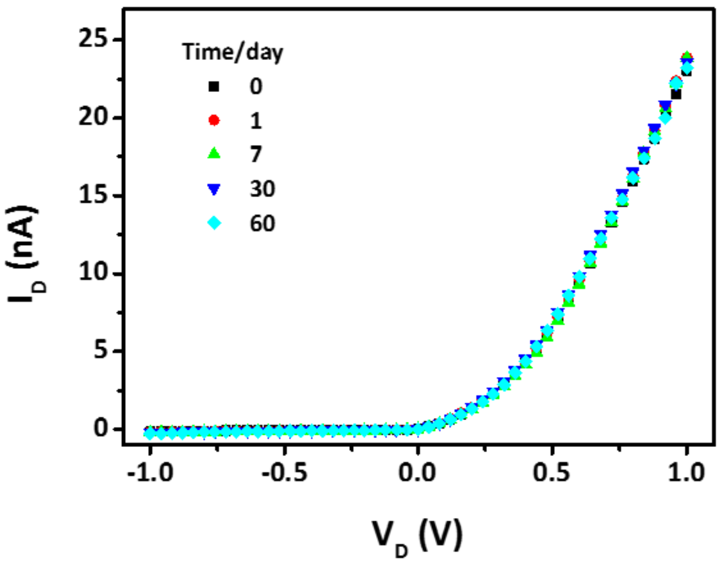

Supplementary Figure 12. I-V characteristic of the CVD monolayer MoS_2 homojunction diode in dark immediately following fabrication and after storing under ambient conditions for 60 days.

Supplementary Table 1. Performance comparison of the homojunction photodiodes in 2D materials by chemical treatment or doping.

Device	Layer thickness	Chemicals	Responsivity (mA/W)	Life time
**Our work: lateral MoS_2**	**monolayer (CVD)**	**PSS**	**~308 (bias = 0)<br>~5120 (bias=1V)**	**2 months**
Lateral MoS_2[3]	few-layer	$AuCl_3$	~0.01(bias = 0)<br>~5070 (bias = 1.5V)	\
Lateral MoS_2[4]	monolayer (CVD)	O_2	~100 (bias = 0)	\
Vertical MoS_2[5]	few-layer	$AuCl_3$/BV	~30 (bias = 0)	\
Lateral BP[6]	few-layer	BV	~211 (bias = 0)<br>~180 (bias = 5 mV)	\
Lateral ReS_2[7]	few-layer	$AuCl_3$	No photovoltaic effect<br>~410 (bias = 2 V)	\
Lateral lnSe[8]	few-layer	Lewis acid	~0.1 (bias = 0)	\
Vertical $MoSe_2$[9]	few-layer	Nb annealing	~120 (bias = 0)<br>~3800 (bias= 1V)	\

Supplementary Note 1

X-ray photoelectron spectroscopy (XPS) characterization of MoS_2 with S-vacancies

X-ray photoelectron spectroscopy data were obtained using an ESCALab250 electron spectrometer from Thermo Scientific Corporation with monochromatic 150 W AlKα radiation. Pass energy for the narrow scan is 30 eV. The base pressure was about 6.5×10^{-10} mbar. The binding energies were referenced to the C1s line at 284.8 eV from alkyl or adventious carbon. For XPS peak analysis and deconvolution, the software Avantage was employed, where Voigt line shapes and an active Shirley background were used for peak fitting. The S/Mo ratios were determined from the integrated areas of the S 2p and Mo 3d peaks factored by their corresponding relative sensitivity factors. The error in the S/Mo ratios was obtained from the peak fitting residuals given by the Avantage software.

The as-grown MoS_2 film was transferred onto a 300nm SiO_2 substrate using the same PMMA-assisted transfer method. The transferred MoS_2 film was treated with PEDOT:PSS solution to heal S vacancies, and then immersed in plenty of DI water to wash the PEDOT:PSS solution for 10 minutes. Further, the residual DI water was dried with nitrogen, finally the sample was dried at 100℃ for 10 min to remove the residual DI water. We observed that the S 2p (Supplementary Fig. 8)and Mo 3d (Fig. 2g) characteristic peaks for of MoS_2 shifted in the XPS spectra because the chemical environment of MoS_2 was changed by the change of sulfur vacancies concentration[10,11]. Besides, To quantify the XPS information, we measured the XPS peak area ratio of S 2p to Mo 3d states for the as-grown and self-healed MoS_2. The value of S:Mo ratio was increased from ~1.67 to ~1.86 by the PSS-induced sulfur vacancies self-healing.

Supplementary Note 2

The electron concentration calculation

Figure 3g indicates that the current of the transistor drops sharply, but the electrode contacts still exhibit a linear relationship. Note that since the 50 μm channel of the MoS_2 transistor is long enough, the contact resistance change between the MoS_2 and Au electrodes is negligible. The channel of the monolayer transistor effectively behaves as a resistor with conductivity $\sigma = q\mu N_D$, and the conductivity can also be calculated using the expression $\sigma = \frac{1}{\rho} = 1/(\frac{dV_D}{dI_D} x \frac{WH}{L})$, where N_D is the electron concentration, L = 50 μm is the channel length, H = 0.65 nm is the channel height, and W = 10 μm is the channel width.

Figure 3f suggests that the conductivity of monolayer MoS_2 after sulfur vacancies self-healing decreased sharply from $8.5x10^{-1}$ to $1.4x10^{-3}$ $\Omega^{-1}cm^{-1}$. From the data presented in Supplementary Figure 8**,** we can extract the low-field field-effect mobility of ~0.96 $cm^2\ V^{-1}\ s^{-1}$ and ~0.26 $cm^2\ V^{-1}\ s^{-1}$ for the as-grown and self-healed MoS_2 using the expression $\mu = \frac{dI_D}{dV_G} \mathrm{x} \frac{\mathrm{L}}{\mathrm{W}V_D C_i}$, where $C_i = 1.1x10^{-4}$ F m^{-2} is the capacitance between the channel and the back gate per unit area ($C_i = \varepsilon_0 \varepsilon_r / d$; $\varepsilon_r = 3.9$; $\mathrm{d} = 300$ nm). Thus, it varies about 643 times for electron concentrations ranging from $5.56x10^{19}$ to $8.65x10^{16}$ cm^{-2}.

In fact, a hopping transport model can be explained the behavior of the mobility decrease of self-healed MoS_2. Electrons in the MoS_2 can transport through the sulfur vacancies by hopping. With this model, the average distance between the sulfur vacancies would increase by PSS-induced SVSH. Therefore, it will make both the hopping probability and mobility decrease.

Supplementary Methods

Construction Process: the monolayer MoS_2 homojunction

Monolayer MoS_2 films were grown on SiO_2/Si substrate by CVD[12]. Subsequently, MoS_2 films were transferred onto the substrates with patterned Poly(3,4-ethylenedioxythiophene):poly(4-styrenesulfonate) (PEDOT:PSS) films by a standard PMMA-based transfer[4]. The specific procedures are as follows: Firstly, the 950k PMMA film was spin-coated on the SiO_2/Si substrate, and then exposed to form square hole by electron beam lithography (EBL). Secondly, PEDOT:PSS electrode was deposited onto the substrate with square holes by spin-coating, subsequently annealed on the hotplate at 120℃ for 15 min to remove water, finally the substrate was immersed into acetone for 12 h to dissolve the residual PMMA film. Thirdly, the as-grown monolayer MoS_2 was transferred onto the substrate in order to form a lateral MoS_2 homojunction. Finally, the device structure was fabricated by depositing Cr/Au electrode.

Instead of conventional photolithography[13], a spin-coating method was designed to carry out the PEDOT:PSS patterning, which can eliminate the residual photoresist film barrier effect of PSS-induced SVSH (Fig.1b). It can also diminish the interface contact resistance induced by the residual photoresist film. The unique core-shell structure PEDOT:PSS provides PSS acid on the surface for healing the sulfur vacancies of MoS_2 (Supplementary Fig.1)[14]. Due to the high conductivity of PEDOT:PSS film, the patterned PEDOT:PSS film also severs as organic electrode. Raman spectrum demonstrates the existence of PEDOT:PSS and MoS_2 film (Supplementary Fig.2), and the difference of ~20 cm^{-1} between the out-of-plane (A_{1g}) and in-plane (E_{2g}^{1}) Raman peaks indicates the single layer thickness of MoS_2 film[15]. The thickness of monolayer MoS_2, PEDOT:PSS and metal electrodes were also confirmed by atomic force microscopy (AFM) image (Supplementary Fig.3)[16].

Supplementary References

1. Li Y, Xu CY, Wang JY, Zhen L. Photodiode-like behavior and excellent photoresponse of vertical Si/monolayer MoS_2 heterostructures. *Scientific reports* **4**, 7186 (2014).
2. Zhang Y, Yan X, Yang Y, Huang Y, Liao Q, Qi J. Scanning Probe Study on the Piezotronic Effect in ZnO Nanomaterials and Nanodevices. *Adv. Mater.* **24**, 4647-4655 (2012).
3. Choi MS*, et al.* Lateral MoS_2 p-n junction formed by chemical doping for use in high-performance optoelectronics. *ACS nano* **8**, 9332-9340 (2014).
4. Lee SY*, et al.* Large Work Function Modulation of Monolayer MoS_2 by Ambient Gases. *ACS nano* **10**, 6100-6107 (2016).
5. Li HM*, et al.* Ultimate thin vertical p-n junction composed of two-dimensional layered molybdenum disulfide. *Nat. Commun.* **6**, 6564 (2015).
6. Yu X, Zhang S, Zeng H, Wang QJ. Lateral black phosphorene P–N junctions formed via chemical doping for high performance near-infrared photodetector. *Nano Energy* **25**, 34-41 (2016).
7. Najmzadeh M, Ko C, Wu K, Tongay S, Wu J. Multilayer ReS_2 lateral p-n homojunction for photoemission and photodetection. *Appl. Phys. Express* **9**, 055201 (2016).
8. Lei S*, et al.* Surface functionalization of two-dimensional metal chalcogenides by Lewis acid-base chemistry. *Nat. Nanotechnol.* **11**, 465-471 (2016).
9. Jin Y, Keum DH, An SJ, Kim J, Lee HS, Lee YH. A Van Der Waals Homojunction: Ideal p-n Diode Behavior in $MoSe_2$. *Adv. Mater.* **27**, 5534-5540 (2015).
10. Li H*, et al.* Activating and optimizing MoS_2 basal planes for hydrogen evolution through the formation of strained sulphur vacancies. *Nat. Mater.* **15**, 364 (2016).
11. Amani M*, et al.* Near-unity photoluminescence quantum yield in MoS_2. *Science* **350**, 1065-1068 (2015).
12. Ji Q, Zhang Y, Zhang Y, Liu Z. Chemical vapour deposition of group-VIB metal dichalcogenide monolayers: engineered substrates from amorphous to single crystalline. *Chem. Soc. Rev.* **44**, 2587-2602 (2015).
13. Taylor PG*, et al.* Orthogonal Patterning of PEDOT:PSS for Organic Electronics using Hydrofluoroether Solvents. *Adv. Mater.* **21**, 2314-2317 (2009).
14. Alemu D, Wei H-Y, Ho K-C, Chu C-W. Highly conductive PEDOT:PSS electrode by simple film treatment with methanol for ITO-free polymer solar cells. *Energy Environ. Sci.* **5**, 9662 (2012).
15. Li H*, et al.* From Bulk to Monolayer MoS_2: Evolution of Raman Scattering. *Adv. Funct. Mater.* **22**, 1385-1390 (2012).
16. Radisavljevic B, Radenovic A, Brivio J, Giacometti V, Kis A. Single-layer MoS_2 transistors. *Nat. Nanotechnol.* **6**, 147-150 (2011).
